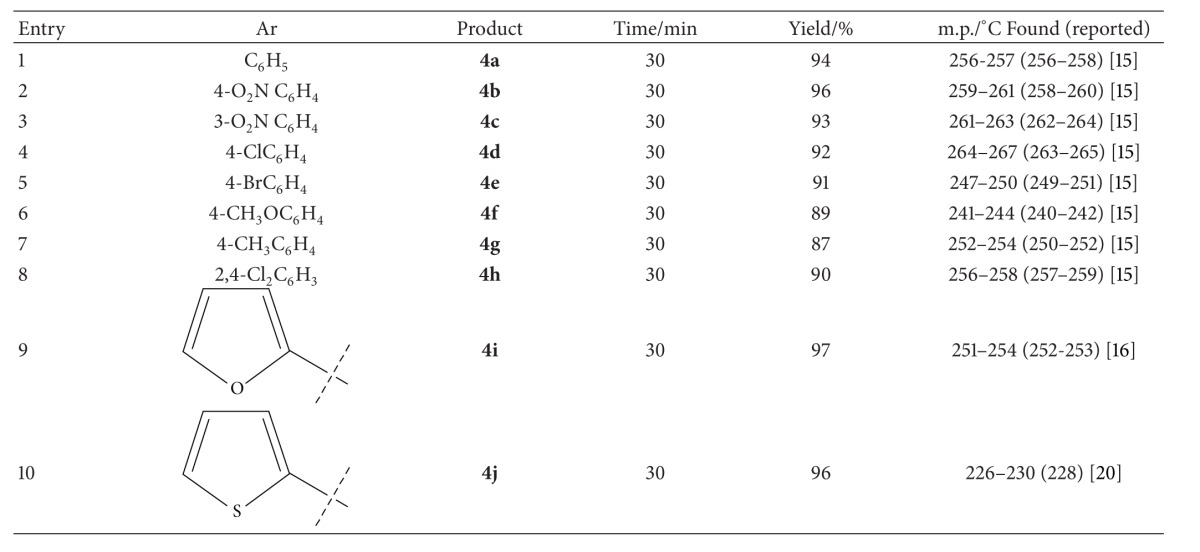# Solvent-Free Green and Efficient One-Pot Synthesis of Dihydropyrano[3,2-*c*]chromene Derivatives

**DOI:** 10.1155/2013/185120

**Published:** 2013-10-24

**Authors:** Shubha Jain, Deepika Rajguru, Balwant S. Keshwal, Aman D. Acharya

**Affiliations:** School of Studies in Chemistry, Vikram University, Ujjain, Madhya Pradesh 456010, India

## Abstract

A rapid, clean, and highly efficient method for synthesis of dihydropyrano[3,2-*c*]chromene derivatives by one-pot, three-component condensation of aromatic aldehydes, malononitrile, and 4-hydroxycoumarin using DABCO as catalyst in solvent-free neat conditions is described. The present method has the advantages of mild reaction conditions, short reaction times, easy isolation of products, and excellent yields.

## 1. Introduction

Multicomponent reactions (MCRs) are very important in organic synthesis due to the formation of carbon-carbon and carbon-hetero atom bonds in one pot [1–3]. Simple procedures, high bond forming efficiency, time and energy saving, and low expenditures are among the advantages of these reactions [4]. Over the past several years, chemists have been aware of the environmental implications of their chemistry. Nowadays, they are trying to develop new synthetic methods, reaction conditions, and uses of chemicals that reduce the risks to humans and the environment. Organic solvents are high on the list of hazardous chemicals because they are used in large amounts and are usually volatile liquids. Therefore, in recent years, solventless organic reactions have attracted great interest. They have many advantages such as high efficiency and selectivity, operational simplicity, low costs, mild reaction conditions, and reduced pollution [5–7]. Pyrano[3,2-*c*]chromenes are a class of important heterocycles with a wide range of biological properties [8] such as spasmolytic, diuretic, anticoagulant, anticancer, and antianaphylactic activity [9]. Moreover, they have been used as cognitive enhancers, for the treatment of neurodegenerative diseases, including Alzheimer's disease, Parkinson's disease, Huntington's disease, amyotrophic lateral sclerosis, AIDS associated dementia, and Down's syndrome as well as for the treatment of schizophrenia and myoclonus [10]. In addition, aminochromene derivatives exhibit a wide spectrum of biological activities including antihypertensive and anti-ischemic behavior [11–13].

 Several methods have been reported for the synthesis of pyrano[3,2-*c*]chromene derivatives. 2-Amino-4-aryl-5-o*x*o-4*H*,5*H*-pyrano[3,2-*c*]chromene-3-carbonitriles have previously been prepared from aromatic aldehydes, malononitrile, and 4-hydroxycoumarin in the presence of organic bases like piperidine or pyridine in an organic solvent, that is, ethanol and pyridine [14]. They have also been prepared in the presence of diammonium hydrogen phosphate (DAHP), (*S*)-proline [15], K_2_CO_3_ under microwave irradiation [16], TBAB [17], MgO [18], H_6_P_2_W_18_O_62_·18H_2_O [19], Hexamethylene tetramine [20], TMGT [21], *N*,*N*,*N*′,*N*′-tetrabromo benzene-1,3-disulfonamide (TBBDA) and poly(*N*,*N*′-dibromo-*N*-ethyl-benzene-1,3-disulfonamide) (PBBS) [22], 3-hydroxypropanaminium acetate (HPAA) [23], 2-hydroxyethylammonium formate [24], [bmim]Br [25], potassium phthalimide-N-oxyl [26], and CuO nanoparticles [27]. However, some of these methods suffer from the serious limitations such as long reaction times, multistep reactions, complex synthetic pathways, and lower product yields. Therefore, the development of milder, faster, and more ecofriendly methods, accompanied with higher yields is needed. 

In recent years, 1,4-diazabicyclo[2.2.2]octane (DABCO) has received considerable attention as an inexpensive, ecofriendly, high reactive, easy to handle, and nontoxic base catalyst for various organic transformations, affording the corresponding products in excellent yields with high selectivity [28, 29]. The reactions are environmentally friendly and the catalyst can be recycled in some cases. In continuation of our interest to further enlarge the application of DABCO as a catalyst [30], here we wish to report one-pot synthesis of pyrano[3,2-*c*]chromene derivatives by the reaction of aromatic aldehydes, malononitrile, and 4-hydroxycoumarin catalyzed by DABCO in solventless conditions.

## 2. Results and Discussion

**Scheme 1:**
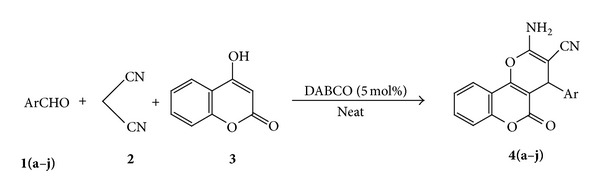


When aromatic aldehyde **1**, malononitrile **2**, and 4-hydroxy coumarin **3** were condensed in the presence of DABCO in solvent-free neat conditions at 100°C; 2-Amino-4-aryl-3-cyano-5-o*x*o-4*H*,5*H*-pyrano[3,2-*c*]chromene derivatives **4** were obtained in good to high yields (Scheme 1).

In our initial study, the reaction of benzaldehyde, malononitrile, and 4-hydroxycoumarin was used as a model reaction to optimize the reaction conditions. First the reaction was conducted in various solvents using DABCO as a catalyst under refluxing conditions and also under solvent-free conditions. As can be seen from Table 1, the best results were obtained in neat. The effect of temperature in solventless conditions was studied by carrying out the reaction at 60, 80, 100, and 120°C. The results from Table 1 (entry 6) showed that 100°C would be the best temperature for all reactions.

Under the optimized reaction conditions, a series of dihydropyrano[3,2-*c*]chromene derivatives **4(a–j)** were synthesized. The results are summarized in Table 2. In all cases, aromatic aldehydes substituted with either electron-donating or electron-withdrawing groups underwent the reaction smoothly and gave the expected products in good to high yields under the same reaction conditions. Moreover, heteroaromatic aldehydes could also be successfully converted to the corresponding heteroaryl substituted pyrano[3,2-*c*]chromenes in excellent yields.

## 3. Conclusion

In summary, a new clean and efficient protocol for the synthesis of pyrano[3,2-*c*]chromene derivatives using DABCO under solvent-free conditions was described. The use of DABCO as a green, nontoxic, nonexplosive, inexpensive, nonvolatile, easy to handle, and thermally stable catalyst with simple experimental and isolation procedure makes it an attractive method for the preparation of these compounds. 

## 4. Experimental

### 4.1. General

All chemicals were purchased from Merck and Sigma-Aldrich as “synthesis grade” and used without further purification. Melting points were determined in open glass capillaries and are uncorrected. ^1^H NMR spectra were obtained at 400 MHz with a Bruker (AVANCE) spectrometer using DMSO-d_6_ as solvent and TMS as an internal standard. Elemental analysis was performed using Carlo Erba-1108 analyzer.

### 4.2. General Procedure for the Synthesis of 2-Amino-4-aryl-5-o*x*o-4*H*,5*H*-pyrano[3,2-c]chromene-3-carbonitriles

Aromatic aldehyde **1** (1 mmoL), malononitrile **2** (1.2 mmoL), 4-hydroxycoumarin **3** (1 mmoL), and DABCO (5 moL%) were mixed thoroughly and heated in a water bath at 100°C for appropriate time. After completion of the reaction (monitored by TLC), the mixture was cooled to room temperature. The solid product was washed with hot water dried and recrystallized from ethanol to give the pure product.

All the compounds were characterized by spectroscopic and physical data which were found to be identical to those described in the literature.

#### 4.2.1. 2-Amino-5-oxo-4-phenyl-4,5-dihydropyrano[3,2-c]chromene-3-carbonitrile ****(4a)****

White Solid, Yield: 94%; m.p.256-257°C (256–258°C) [15]; Anal. Calcd. for C_19_H_12_N_2_O_3_: C, 72.15; H, 3.82; N, 8.86%. Found: C, 72.28; H, 3.61; N, 8.65%; ^1^H-NMR (400 MHz, DMSO-d_6_, **δ**/ppm): 4.16 (1H, *s*, CH), 6.34 (2H, *s*, amino group), 7.35–7.44 (5H, *m*, ArH), 7.53–7.58 (2 H, *m*, ArH), 7.65–7.71 (2H, *m*, ArH); ^13^C-NMR (400 MHz, DMSO-d_6_, **δ**/ppm): 53.27, 103.62, 113.38, 115.83, 116.01, 121.32, 123.14, 123.53, 124.85, 125.08, 127.12, 128.37, 141.35, 152.78, 154.84, 158.19, 159.65.

#### 4.2.2. 2-Amino-4-(4-nitrophenyl)-5-oxo-4,5-dihydropyrano[3,2-c]chromene-3-carbonitrile ****(4b)****

Pale Yellow Solid, Yield: 96%; m.p. 259–261°C (258–260°C) [15]; Anal. Calcd. for C_19_H_11_N_3_O_5_: C, 63.16; H, 3.07; N, 11.63%. Found: C, 63.29; H, 3.19; N, 11.95%; ^1^H-NMR (400 MHz, DMSO-d_6_, **δ**/ppm): 4.76 (1H, *s*, CH), 6.43 (2H, *s*, amino group), 7.20–7.22 (2H, *dd*, *J*_*a*_ = 3.9 Hz, *J*_*b*_ = 1.0 Hz, ArH), 7.27 (1H, *s*, ArH), 7.42 (1H, *d*, *J* = 3.6 Hz, ArH), 7.63–7.65 (1H, *dd*, *J*_*a*_ = 4.2 Hz, *J*_*b*_ = 0.9 Hz, ArH), 7.69 (1H, *d*,* J* = 3.7 Hz, ArH), 7.76 (2H, *d*,* J* = 1.2 Hz, ArH); ^13^C-NMR (400 MHz, DMSO-d_6_, **δ**/ppm): 57.34, 105.83, 112.56, 115.91, 116.73, 119.26, 122.06, 123.12, 125.49, 126.64, 129.03, 138.23, 148.39, 152.84, 153.17, 158.35, 159.46.

#### 4.2.3. 2-Amino-4-(3-nitrophenyl)-5-oxo-4,5-dihydropyrano[3,2-c]chromene-3-carbonitrile ****(4c)****

White Solid, Yield: 93%; m.p. 261–263°C (262–264°C) [15]; Anal. Calcd. for C_19_H_11_N_3_O_5_: C, 63.16; H, 3.07; N, 11.63%. Found: C, 63.28; H, 3.17; N, 11.96%; ^1^H-NMR (400 MHz, DMSO-d_6_, **δ**/ppm): 4.26 (1H, *s*, CH), 6.32 (2H, *s*, amino group), 7.15–7.24 (3H, *m*, ArH), 7.46 (2H, *d*,* J* = 7.6 Hz, ArH), 7.90 (1H, *d*,* J* = 2.0 Hz, ArH), 8.33 (1H, *d*,* J* = 7.6 Hz, ArH), 8.87 (1H, *s*, ArH); ^13^C-NMR (400 MHz, DMSO-d_6_, **δ**/ppm): 55.37, 107.58, 113.35, 115.19, 116.24, 119.73, 122.52, 123.65, 125.54, 128.73, 129.46, 135.48, 148.36, 152.11, 153.72, 158.02, 159.78.

#### 4.2.4. 2-Amino-4-(4-chlorophenyl)-5-oxo-4,5-dihydropyrano[3,2-c]chromene-3-carbonitrile ****(4d)****

White Solid, Yield: 92%; m.p. 264–267°C (263–265°C) [15]; Anal. Calcd. for C_19_H_11_ClN_2_O_3_: C, 65.06; H, 3.16; N, 7.99%. Found: C, 65.35; H, 3.26; N, 7.73%; ^1^H-NMR (400 MHz, DMSO-d_6_, **δ**/ppm): 4.72 (1H, *s*, CH), 6.74 (2H, *s*, amino group), 7.22 (1H, *t*,* J* = 4.6 Hz, ArH), 7.70–7.73 (3H, *m*, ArH), 7.84 (2H, *d*,* J* = 8.6 Hz, ArH), 8.36 (2H, *d*,* J* = 8.6 Hz, ArH); ^13^C-NMR (400 MHz, DMSO-d_6_, **δ**/ppm): 54.72, 106.43, 112.78, 115.62, 116.58, 119.32, 121.09, 123.45, 124.64, 125.92, 128.53, 134.32, 138.10, 152.18, 152.97, 158.49, 159.56.

#### 4.2.5. 2-Amino-4-(4-bromophenyl)-5-oxo-4,5-dihydropyrano[3,2-c]chromene-3-carbonitrile ****(4e)****

White Solid, Yield: 91%; m.p. 247–250°C (249–251°C) [15]; Anal. Calcd. for C_19_H_11_BrN_2_O_3_: C, 57.74; H, 2.81; N, 7.09%. Found: C, 57.53; H, 2.96; N, 7.17%; ^1^H-NMR (400 MHz, DMSO-d_6_, **δ**/ppm): 4.36 (1H, *s*, CH), 6.60 (2H, *s*, amino group), 7.44 (2H, *d*,* J* = 3.5 Hz, ArH), 7.77 (1H, *d*,* J* = 1.7 Hz, ArH), 7.82–7.84 (3H, *m*, ArH), 8.36–8.38 (2H, *dd*, *J*_*a*_ = 4.9 Hz, *J*_*b*_ = 1.9 Hz, ArH); ^13^C-NMR (400 MHz, DMSO-d_6_, **δ**/ppm): 55.43, 105.57, 112.58, 115.23, 116.62, 119.82, 121.08, 123.42, 124.78, 126.56, 127.58, 136.45, 139.34, 152.49, 152.84, 157.37, 159.89.

#### 4.2.6. 2-Amino-4-(4-methoxyphenyl)-5-oxo-4,5-dihydropyrano[3,2-c]chromene-3-carbonitrile ****(4f)****

White Solid, Yield: 89%; m.p. 241–244°C (240–242°C) [15]; Anal. Calcd. for C_20_H_14_N_2_O_4_: C, 69.36; H, 4.07; N, 8.09%. Found: C, 68.92; H, 4.20; N, 8.19%; ^1^H-NMR (400 MHz, DMSO-d_6_, **δ**/ppm): 3.76 (3H, *s*, OCH_3_), 5.13 (1H, *s*, CH), 6.36 (2H, *s*, amino group), 6.65-6.66 (2H, *q*,* J* = 1.7 Hz, ArH), 7.41 (2H, *d*,* J* = 3.3 Hz, ArH), 7.57 (2H, *m*, ArH), 7.75 (2H, *d*,* J* = 1.5 Hz, ArH); ^13^C-NMR (400 MHz, DMSO-d_6_, **δ**/ppm): 52.95, 57.66, 103.90, 112.96, 116.26, 117.39, 119.28, 122.49, 123.72, 124.32, 125.26, 126.51, 132.42, 138.10, 152.01, 152.95, 158.24, 159.53.

#### 4.2.7. 2-Amino-5-oxo-4-p-tolyl-4,5-dihydropyrano[3,2-c]chromene-3-carbonitrile ****(4g)****

White Solid, Yield: 87%; m.p. 252–254°C (250–252°C) [15]; Anal. Calcd. for C_20_H_14_N_2_O_3_: C, 72.72; H, 4.27; N, 8.48%. Found: C, 72.46; H, 4.19; N, 8.58%; ^1^H-NMR (400 MHz, DMSO-d_6_, **δ**/ppm): 2.10 (3H, *s*, CH_3_), 4.58 (1H, *s*, CH), 6.73 (2H, *s*, amino group), 7.22–7.24 (4H, *m*, ArH), 7.70-7.71 (2H, *dd*, *J*_*a*_= 2.7 Hz, *J*_*b*_ = 1.0 Hz, ArH), 7.74–7.76 (2H, *dd*, *J*_*a*_ = 4.2 Hz, *J*_*b*_ = 1.0 Hz, ArH); ^3^C-NMR (400 MHz, DMSO-d_6_, **δ**/ppm): 13.95, 30.57, 55.59, 101.64, 111.65, 112.92, 116.24, 118.88, 122.44, 124.20, 132.47, 146.91, 150.86, 151.93, 152.08, 153.90, 158.74, 159.35.

#### 4.2.8. 2-Amino-4-(2,4-dichlorophenyl)-5-oxo-4,5-dihydropyrano[3,2-c]chromene-3-carbonitrile ****(4h)****

White Solid, Yield: 90%; m.p. 256–258°C (257–259°C) [15]; Anal. Calcd. for C_19_H_10_Cl_2_N_2_O_3_: C, 59.24; H, 2.62; N, 7.27%. Found: C, 59.51; H, 2.49; N, 7.48%; ^1^H-NMR (400 MHz, DMSO-d_6_, **δ**/ppm): 4.24 (1H, *s*, CH), 6.31 (2H, *s*, amino group), 6.66 (1H, *d*,* J* = 3.1 Hz, ArH), 6.71 (1H, *t*,* J* = 1.4 Hz, ArH), 7.30 (1H, *d*,* J* = 3.2 Hz, ArH), 7.59 (2H, *m*, ArH), 7.85 (1H, *s*, ArH), 7.95 (1H, *d*,* J* = 0.9 Hz, ArH); ^13^C-NMR (400 MHz, DMSO-d_6_, **δ**/ppm): 28.38, 57.99, 104.05, 113.36, 115.97, 116.19, 119.36, 120.50, 122.40, 123.38, 124.23, 125.34, 127.15, 132.26, 135.03, 151.95, 152.77, 158.15, 159.53.

#### 4.2.9. 2-Amino-4-(furan-2-yl)-5-oxo-4,5-dihydropyrano[3,2-c]chromene-3-carbonitrile ****(4i)****

Brown Solid, Yield: 97%; m.p. 251–254°C (252-253°C) [16]; Anal. Calcd. for C_17_H_10_N_2_O_4_: C, 66.67; H, 3.29; N, 9.15%. Found: C, 66.95; H, 3.07; N, 9.27%; ^1^H-NMR (400 MHz, DMSO-d_6_, **δ**/ppm): 3.92 (1H, *s*, CH), 6.30 (2H, *s*, amino group), 6.79–6.81 (1H, *dd*, *J*_*a*_ = 4.9 Hz, *J*_*b*_ = 1.8 Hz, furan ring), 7.08–7.12 (3H, *m*, furan ring+ArH), 7.38–7.41 (1H, *dd*, *J*_*a*_ = 3.4 Hz, *J*_*b*_ = 1.6 Hz, ArH), 7.75 (1H, *d*,* J* = 2.5 Hz, ArH), 7.86 (1H, *d*,* J* = 4.7 Hz, ArH); ^13^C-NMR (400 MHz, DMSO-d_6_, **δ**/ppm): 30.49, 55.87, 101.64, 106.16, 106.98, 111.55, 112.90, 116.17, 118.86, 122.44, 124.11, 150.92, 151.67, 152.05, 153.94, 158.71, 159.50. 

#### 4.2.10. 2-Amino-5-oxo-4-(thiophen-2-yl)-4,5-dihydropyrano[3,2-c]chromene-3-carbonitrile ****(4j)****

White Solid, Yield: 96%; m.p. 226–230°C (228°C) [20]; Anal. Calcd. for C_17_H_10_N_2_O_3_S: C, 63.34; H, 3.13; N, 8.69; S, 9.95%. Found: C, 63.53; H, 3.24; N, 8.37; S, 9.68%; ^1^H-NMR (400 MHz, DMSO-d_6_, **δ**/ppm): 4.94 (1H, *s*, CH), 6.54 (2H, *s*, amino group), 6.98 (1H, *m*, thiophene ring), 7.20–7.22 (2H, *dd*, *J*_*a*_ = 3.8 Hz, *J*_*b*_ = 1.3 Hz, thiophene ring), 7.65–7.67 (2H, *dd*, *J*_*a*_ = 4.2 Hz, *J*_*b*_ = 1.0 Hz, ArH), 7.69-7.70 (2H, *dd*, *J*_*a*_ = 2.6 Hz, *J*_*b*_ = 1.1 Hz, ArH); ^13^C-NMR (400 MHz, DMSO-d_6_, **δ**/ppm): 31.94, 57.84, 103.97, 116.22, 118.94, 122.51, 124.27, 124.58, 124.67, 127.72, 132.52, 138.41, 140.90, 152.95, 153.84, 158.40, 159.49.

## Figures and Tables

**Table 1:** DABCO catalyzed synthesis of **4a** in different reaction conditions.

Entry	Solvent	*T*/°C	Time/h	Yield^a^/%
1	EtOH	Reflux	2	85
2	CH_2_Cl_2_	Reflux	6	54
3	CH_3_CN	Reflux	4	73
4	THF	Reflux	4	62
5	H_2_O	Reflux	1.5	87
6	—	100	0.5	94
7	—	60	2	65
8	—	80	1	76
9	—	120	0.5	92
10^b^	—	100	2	65
11^c^	—	100	0.5	95

^a^Isolated yield.

^b^1 mol% of catalyst was used.

^c^10 mol% of catalyst was used.

**Table 2:** Synthesis of 2-Amino-4-aryl-3-cyano-5-o*x*o-4*H*,5*H*-pyrano[3,2-*c*]chromenes in solvent-free neat conditions using DABCO (5 mol%) as catalyst.